# Integration of Lipidomics and Transcriptomics Reveals the Efficacy and Mechanism of Qige Decoction on NAFLD

**DOI:** 10.1155/2022/9739032

**Published:** 2022-11-21

**Authors:** Simin Fan, Zunming Zhou, Jintong Ye, Yanfang Li, Keer Huang, Xuehong Ke

**Affiliations:** ^1^First Clinical School of Guangzhou University of Chinese Medicine, Guangzhou 510410, Guangdong, China; ^2^Lingnan Medical Research Center, Guangzhou University of Chinese Medicine, Guangzhou 510410, Guangdong, China; ^3^The First Affiliated Hospital of Guangzhou University of Chinese Medicine, Guangzhou 510405, Guangdong, China; ^4^Postdoctoral Research Station of Guangzhou University of Chinese Medicine, Guangzhou 510405, Guangdong, China

## Abstract

The prevalence of nonalcoholic fatty liver disease (NAFLD) is increasing as obesity and diabetes become more common. There are no drugs approved for NAFLD yet. Qige decoction (QGD), a traditional Chinese medicine (TCM) formula, is used for NAFLD and hyperlipidemia treatment in TCM and has shown hypolipidemic and hepatoprotective effects. This study tried to interpret the pharmacology and molecular mechanisms of QGD in NAFLD rats. Firstly, the therapeutic effects of QGD on high-fat diet (HFD)-induced NAFLD rats were evaluated. Then, integration of lipidomics and transcriptomics was conducted to explore the possible pathways and targets of QGD against NAFLD. QGD at low dosage (QGL) administration reduced serum total cholesterol (TC), triglyceride (TG), and low-density lipoprotein cholesterol (LDL-C) (*P* < 0.05). Liver histopathology indicated that QGL could alleviate hepatic steatosis. The main differential lipids (DELs) affected by QGD were glycerolipids. KEGG enrichment analysis suggested that the main pathways by which QGD improved NAFLD may be cholesterol metabolism, glycerolipid metabolism, and insulin resistance. Transcriptome sequencing identified 179 upregulated and 194 downregulated mRNAs after QGD treatment. An interaction network based on DELs and differential genes (DEGs) suggested that QGD inhibited hepatic steatosis mainly by reducing hepatic insulin resistance and triglyceride biosynthesis via the PPP1R3C/SIK1/CRTC2 and PPP1R3C/SIK1/SREBP1 signal axis, respectively. These findings indicated that QGD could protect against NAFLD induced by HFD. The improvement of hepatic insulin resistance and the reduction of triglyceride biosynthesis might be the potential mechanisms.

## 1. Introduction

Nonalcoholic fatty liver disease (NAFLD) comprises a wide disease spectrum ranging from steatosis to inflammation to fibrosis [1]. NAFLD, regarded as a hepatic characteristic of the metabolic syndrome (MS) [2], has become more prevalent with the increased prevalence of obesity, diabetes, and MS. The global prevalence of NAFLD is estimated at ∼25% [3], contributing to a growing economic burden [4]. NAFLD-related adverse hepatic outcomes may include cirrhosis, hepatocellular carcinoma, and liver failure. It is predicted to be the primary cause of liver transplants in ten years [5]. In addition, the main extrahepatic-related adverse outcomes are increased cardiovascular disease and malignancy [6].

The liver is essential for glucose and lipid metabolism. Therefore, the onset and development of NAFLD are closely related to disorders of glucose and lipid metabolism, especially insulin resistance. Hepatic insulin resistance has been shown to promote hepatic de novo lipogenesis (DNL) and gluconeogenesis while decreasing glycogen synthesis, which was referred to as “selective insulin resistance” [7]. Hepatic insulin resistance was thought to direct glucose into lipogenesis pathways and further promote the development of NAFLD [8]. Meanwhile, increased intrahepatic ectopic lipids may promote hepatic insulin resistance [9]. A possible mechanism of lipid-induced hepatic insulin resistance is that hepatic diacylglycerol deposition inhibits IRTK activation while promoting PKC activation [10, 11].

Although there has been steady progress in identifying therapeutic targets and promoting drug development. Improving metabolism is a potentially effective method for the treatment of NAFLD with approaches to improving insulin sensitivity, decreasing the delivery of metabolic substrates to the liver and hepatic DNL, or facilitating its safe disposal. For example, obeticholic acid (OCA), an activator of the farnesol X receptor, can improve insulin sensitivity and show anti-inflammatory and antifibrotic activity in nonalcoholic steatohepatitis (NASH) patients [12]. Acyl-CoA carboxylase (ACC) inhibitors may potentially ameliorate NASH by reducing DNL [13]. PPAR*α*/*δ* agonists possess dual activity on both PPAR*α* and PPAR*δ* receptors. The PPAR*α* component increases fatty acid *β* oxidation and the PPAR*δ* component attenuates inflammation [14]. However, these drugs undergoing research have some limitations. OCA could cause both pruritus and elevated low-density lipoprotein cholesterol (LDL-C) [15]. ACC inhibitors promote hypertriglyceridemia [13]. In short, there are great challenges in drug development, and no agents have been approved yet.

In China, traditional Chinese medicine (TCM) has a history of 5000 years and extensive use. Many TCMs have shown significant efficacy in the treatment of NAFLD, Silibinin capsules [16], Hugan Qingzhi tablet [17], etc. Qige decoction (QGD, a TCM formula) consists of a 6 : 2 : 1 ratio of *Astragalus mongholicus* Bunge (Huangqi), *Pueraria montana* var. *Lobata* (Willd.) Maesen and S. M. Almeida ex Sanjappa and Predeep (Gegen), and *Citrus* × *aurantium L*. (Chenpi). The first two are drug pairs that were first recorded in “ZhengzhiHuibu” by Li Yongcui (1687 AD). Huangqi-Gegen drug pair possesses a wide use to alleviate NAFLD in clinical practice. Modern pharmaceutical research expressed that it could improve insulin resistance [18]. Chenpi, according to the theory of chief, deputy, assistant, and envoy that is highly respected by TCM formulation, is often used as an assistant medicine to strengthen the therapeutic effect of chief and deputy medicines. Moreover, our previous research showed that Chenpi could alleviate hyperlipidemia mainly through taurine and hypotaurine metabolism, fatty acid biosynthesis, and arginine and proline metabolism pathways [19]. QGD can strengthen the spleen, benefit qi, dispel dampness, and clear heat. It is suitable for NAFLD, which is believed to be due to spleen deficiency leading to damp encumbrance in view of TCM pathophysiology. We have found that QGD could ameliorate CCl4-induced liver injury by activating the Nrf2 pathway as an antioxidant [20]. Herein, we tried to interpret the pharmacology of QGD on NAFLD and explore its potential mechanism via the integration of lipidomics and transcriptomics.

Lipidomics focuses on lipid metabolism and has been used to reveal the lipidome changes mediated by disease effects, especially metabolic diseases, such as NAFLD and MS [21]. Therefore, lipidomics was used to identify and quantify the hepatic lipidome to find out the changes in lipid metabolites during NAFLD and QGD administration. Transcriptomics can identify thousands of differentially genes (DEGs) to show the overall gene expression profile during NAFLD and QGD administration. The metabolite changes were tightly associated with gene transcription regulation. Integrating gene expression profiles and metabolic profiles was considered a novel approach to understanding the complicated regulatory network of genetic and metabolic pathways [22]. Hence, we also conducted a correlation analysis between lipid metabolites and gene expression to preliminarily explore the targets and pathways of QGD in the treatment of NAFLD.

## 2. Material and Method

### 2.1. Drugs Preparation

The plant compositions of QGD are shown in Table 1. All medicinal plants of QGD were simmered for 30 min three times after boiling (with distilled water, 1 : 20, 1 : 15, and 1 : 15 V/V, respectively). After filtration, the mixed solution was concentrated to 1 g raw herb/mL.

Atorvastatin calcium tablet (ATO, Cat: 4309784) purchased from Pfizer Pharmaceuticals Co., Ltd (USA) was dissolved in 0.5% CMC-NA suspension to prepare 0.5 mg/mL Atorvastatin calcium suspension.

### 2.2. Animals and Tissue Collection

Fifty male Sprague–Dawley rats, VAH/SPF level, weighing 200 ± 20 g, were purchased from Guangdong Medical Laboratory Animal Centre (Guangzhou, China) and raised in an air-conditioned room at 21 ± 0.3°C with a regular 12 h light/dark photoperiod and 60 ± 5% relative humidity. Food and water were freely available. After 7 days of acclimation, all rats were randomly assigned to the control (CON) group, the model (MOD) group, the ATO group (5 mg/kg ATO), the QGD at low dosage (QGL) group (5 g crude drug/kg QGD, equivalent to 1 time the usual clinical therapeutic dose for QGD), and the QGD at high dosage (QGH) group (10 g crude drug/kg QGD, equivalent to 2 times the usual clinical therapeutic dose for QGD) (*n* = 10). CON rats were fed with a normal diet (Guangzhou, China, crude protein ≥1.8%, calcium 1%–1.8%, crude fiber ≤5%, crude Ash ≤8%, total phosphorus 0.6%–1.2%, crude fat ≥4%, sodium chloride 0.5%–0.9%, lysine 0.82%, and water ≤100), while other groups were fed with a HFD (Guangzhou, China, 20% sucrose, 15% lard, 1.2% cholesterol, 0.2% sodium cholate, 10% casein, 0.6% calcium hydrogen phosphate, 0.4% stone powder, 0.4% premix, and 52.2% base feed) for 20 days. Body weight was recorded every day. All rats were anesthetized by intraperitoneal injection of sodium pentobarbital (30 mg/kg) on day 20. Blood was collected from the abdominal aorta and centrifuged at 3000 g for 10 min to get serum for subsequent biochemical analysis. The livers were removed from each animal. Then, 200 mg of samples excised from the left lobe of the liver were stored at −80°C for lipidomics (*n* = 6), while another 200 mg of samples excised from the left lobe were used for transcriptomics (*n* = 3). The remaining liver lobes were subjected to hematoxylin-eosin (HE) and oil red O staining. All protocols and studies involving animals have been approved by the Animal Ethics Committee of the Guangzhou University of Chinese Medicine (No. TCMF1-2019011).

### 2.3. Serum Metabolites

Serum total cholesterol (TC), triglyceride (TG), high-density lipoprotein cholesterol (HDL-C), and LDL-C were detected by using a kit from Beijing Jiuqiang Biological Company (Beijing, China).

### 2.4. HE and Oil Red O Staining

Liver histology was assessed by HE staining, while lipid accumulation in livers was observed by oil red O staining. The liver tissue was fixed with 4% paraformaldehyde, then embedded in paraffin, cut into slices of approximately 4 *µ*m and stained with HE. The frozen liver tissues were put in the optimal cutting temperature compound (OCT), cut into 8–10 *μ*m thick, and stained using oil red O solution. Images were obtained under an Olympus BX54 inverted microscope (Tokyo, Japan) at 400× magnification.

### 2.5. Sample Preparation

Since pharmacological results showed that the effects of the QGL group were superior to those of the QGH group, the QGL group was included in further lipidomics and transcriptomic analysis. Six rats in the CON, MOD, and QGL groups were randomly selected and included in the widely-targeted lipidomics analysis. After thawing on ice, 20 g of liver tissues were homogenized with 1 mL lipid extraction reagent (methyl tertiary-butyl ether: methanol = 3 : 1, V/V, containing internal standard mixture). Samples were mixed on a vortexer for 15 min and 200 uL distilled water was added and then mixed for 1 min. After centrifugation at 12000 g for 10 min at 4°C, the supernatant was transferred and concentrated. The mixture of 10% acetonitrile and 90% isopropanol with 0.1% formic acid and 10 mmol/L ammonium formate was added to the supernatant for the UPLC-MS/MS analysis.

### 2.6. UPLC-MS/MS Detection

The liver lipid profiling was performed on ultra-performance liquid chromatography (UPLC, ExionLC AD, sciex, USA) coupled with QTRAP tandem mass spectrometry (sciex, USA). Chromatographic separations involved the use of a Thermo Accucore™ C30 column (2.6 *μ*m, 2.1*∗*100 mm) (Thermo Fisher Scientific, USA) with a 20 min gradient. Mobile phases were A (60% acetonitrile in water, 0.1% formic acid, 10 mmol/L ammonium formate) and B (10% acetonitrile, 90% isopropanol, 1% formic acid, and 10 mmol/L ammonium formate). The UPLC conditions were as follows: flow rate was 0.35 mL/min, column temperature was 45°C, and injection volume was 2 *μ*L. The solvent gradient program was: 0–2 min 20–30% B; 2–4 min 30–60% B; 4–9 min 60–85% B; 9–14 min 85–90% B; 14–15.5 min 90–95% B; 15.5–17.3 min, hold 95% B; 17.4, 20% B; 17.4–20 min, hold 20% B. The mass spectrometer was equipped with an electrospray ionization source. Data acquisition was performed with MRM scanning in positive ion mode (5.5 kV). The detailed parameters of the mass spectrometer's ion source were as follows: CUR = 35 psi, GS1 = 45 psi, GS2 = 55 psi, IS = 5500 V, CAD = MEDIUM, and TEMP = 500°C. The mass spectral data were analyzed by Software Analyst 1.6.3. To visualize the metabolic alterations between groups, principle component analysis (PCA) was carried out with *R* 3.5.0 and orthogonal partial least-squares discriminant analysis (OPLS-DA) with SIMCA-P14.1 software. Significantly regulated lipids among groups were determined by VIP ≥ 1 and absolute Log2FC (fold change) ≥1. Metabolic pathway analysis was performed by metabolite set enrichment analysis.

### 2.7. Transcriptome Sequencing

The total RNA of liver tissues was isolated with the RNAiso Plus Reagent (Takara Biotech, Kyoto, Japan). The degradation and contamination of RNA were monitored by agarose gel electrophoresis. After the cDNA library was constructed, Qubit 2.0 (Life Technologies, CA, USA) was used for initial quantification and Agilent 2100 (Agilent Technologies, CA, USA) for quality testing.

After quality testing, sequencing was performed on the Illumina HiSeq platform. Clean reads were obtained by filtering raw data, checking sequencing error ratio, and GC level distribution. Sequence alignment was performed with the designated reference genome using hisat2 2.1.0 to obtain mapped data. The FPKM of each gene was calculated according to the length of the gene and the number of reads mapped to that gene. DEGs were analyzed with the DESeq2 R package (version 1.22.2). The Kyoto Encyclopedia of Genes and Genomes (KEGG) was used for differential gene annotation.

### 2.8. Integration of Lipidomics and Transcriptomic Analysis

The correlation analysis of the DEGs and differential lipids (DELs) was carried out via calculating Pearson's correlation coefficient (PCC) using *R* 1.22.2. Then the DEGs and DELs with PCC >0.8 were screened. The correlation coefficient cluster heat map was conducted with *R* 1.22.2, and the Cytoscape was adopted to show the relationship between DEGs and DELs. The DEGs and DELs were mapped to the KEGG pathway map. Enrichment analysis was performed to obtain the pathway which a *p* value < 0.05 and draw a histogram.

### 2.9. Statistical Analysis

The results of blood lipid and body weight were analyzed by the SPSS 25.0 software (IBM Corporation, Armonk, NY, USA) and expressed as the mean ± standard deviation. A one-way variance analysis was adopted when both normality and homogeneity were met; otherwise, a nonparametric test was used. The statistical significance was defined as *P* < 0.05, and *P* < 0.01 was considered highly significant.

## 3. Results

### 3.1. QGD Protected Rats against HFD-Induced Hepatic Steatosis

**Figure 1:**
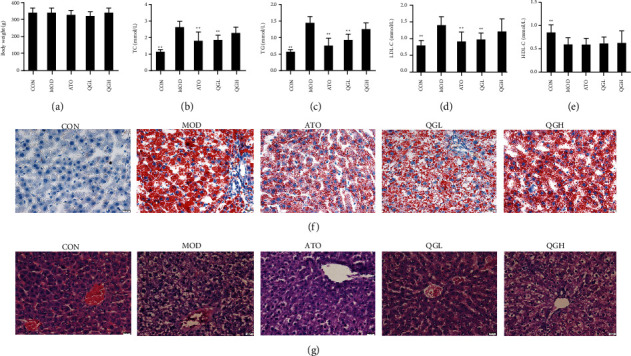
QGD protected rats against hepatic steatosis in HFD rats. (a) Effects of QGD on body weight (g) at the end of the intervention. (b) Effects of QGD on TC. (c) Effects of QGD on TG. (d) Effects of QGD on LDL-C. (e) Effects of QGD on HDL-C. (f) Oil red O staining of liver tissues (400×). (g) HE staining of liver tissues (400×). Notes: ^*∗*^*P* < 0.05, ^*∗∗*^*P* < 0.01 versus MOD group.

Body weight showed no significant differences among the groups (*P* > 0.05) (Figure 1(a)). Serum TC, TG, and LDL-C levels were significantly increased in the MOD group after 20 days of HFD administration, while HDL-C was significantly decreased (*P* < 0.01) (Figures 1(b)–1(e)). Compared to the MOD group, both QGL and ATO could lower serum TC, TG, and LDL-C (*P* < 0.05), while the reductions of these indexes were not significant in the QGH group (*P* > 0.05). There were no significant differences in HDL-C levels among the MOD, ATO, QGL, and QGH groups (*P* > 0.05). HE staining and oil red O staining demonstrated obvious lipid deposition in the livers of the MOD group, while ATO and QGL could significantly decrease the size and number of hepatic lipid droplets. However, QGL improved lipid deposition more significantly than QGH (Figures 1(f) and 1(g)).

### 3.2. QGD Modulated Hepatic Lipid Profiles

**Figure 2:**
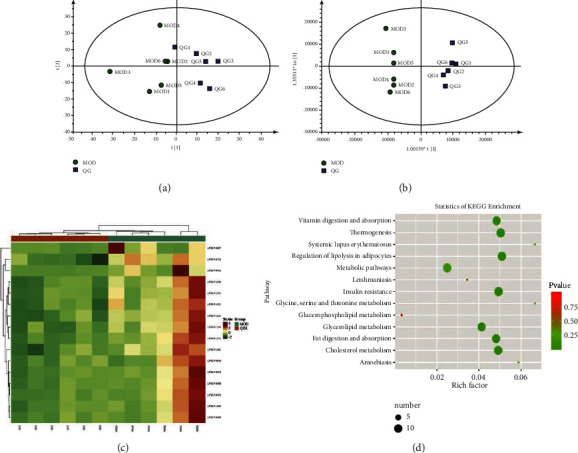
QGD modulates liver lipids profiles (QGL vs MOD, *n* = 6). (a) Plots of PCA. (b) Plots of OPLS-DA analysis. (c) Heatmap of DELs. (d) Dot plot of KEGG enrichment for liver lipids profiles.

To analyze the effects of QGD on hepatic lipid profiles of NAFLD rats, PCA and OPLS-DA models were adopted to compare the liver samples obtained from the QGL and MOD groups because of the more significant improvement of liver histopathology and biochemical parameters in the QGL group than the QGH group. Although PCA analysis presented a partial overlap of the MOD and QGL groups (Figure 2(a)). The OPLS-DA score plot (Figure 2(b)) showed that the two groups were separated into different clusters, which suggested that the hepatic metabolic phenotypes between the MOD and QGL groups were different. 16 DELs identified between the MOD and QGL groups, which suggested QGD mainly affected glycerolipids (13 in total: triglycerides) and fatty acyls (2 in total: acylcarnitine), are shown in Figure 2(c) and details are shown in Table 2. KEGG enrichment (Figure 2(d)) suggested cholesterol metabolism, fat digestion and absorption, glycerolipid metabolism, insulin resistance, regulation of lipolysis in adipocytes, thermogenesis and vitamin digestion and absorption were the main pathways. Among these pathways, cholesterol metabolism, glycerolipid metabolism, and insulin resistance are closely related to NAFLD.

### 3.3. Hepatic Transcriptomic Expression Alteration

**Figure 3:**
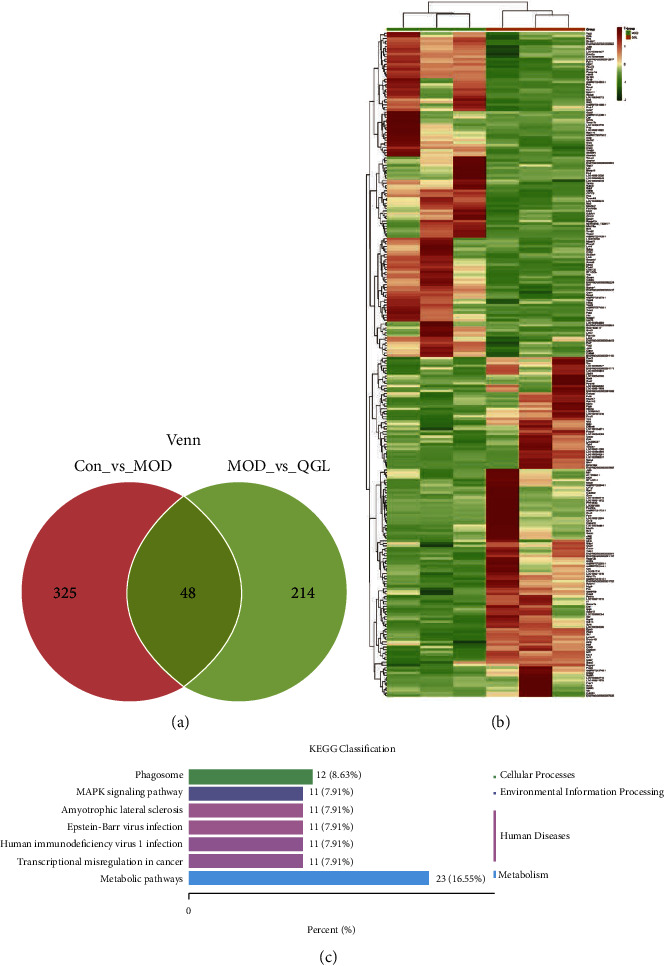
Transcriptomic data analysis (QGL vs MOD, *n* = 3). (a) Wayne figure of DEGs among groups. (b) The cluster heatmap of 262 DEGs between the QGL and MOD groups. (c) The KEGG classification analysis of 262 DEGs between the QGL and MOD groups.

In transcriptome sequencing, the DEGs were determined with the screening criteria of |log2FC| > 1 and *P* < 0.05. 373 DEGs (179 upregulated and 194 downregulated) were identified between the CON and MOD groups. After QGL administration, 262 genes in the MOD group were significantly regulated (134 upregulated and 128 downregulated) (Figure 3(a)). A cluster heatmap of 262 DEGs between the MOD and QGL groups is shown in Figure 3(b). An analysis of KEGG gene annotation classification revealed that 23 (16.55%) DEGs concentrated on the functional categories were related to metabolic pathway (Figure 3(c)).

### 3.4. Integrated Analysis of Lipidomics and Transcriptomics

**Figure 4:**
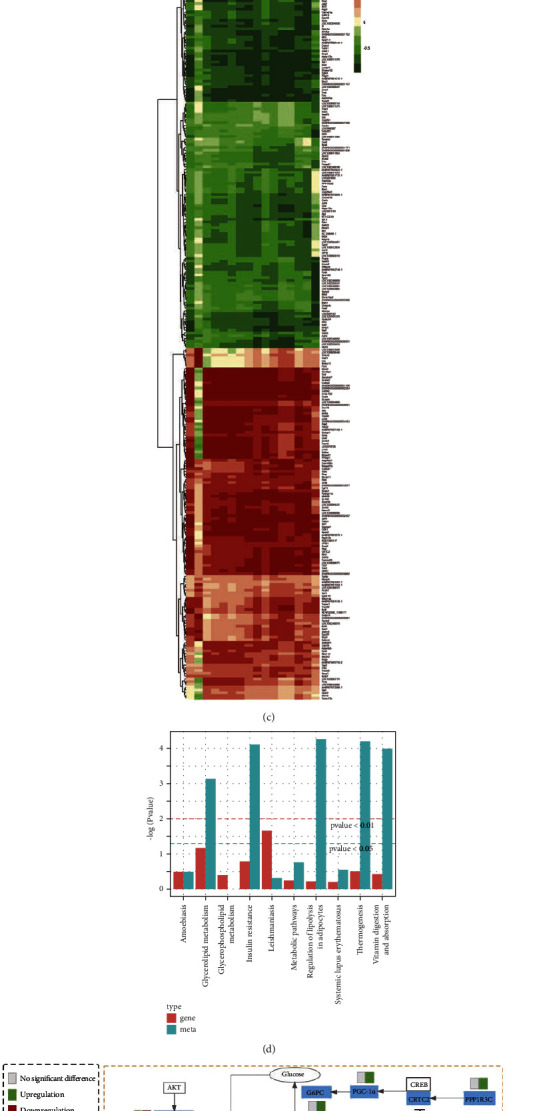
Integrated analysis of lipidomics and transcriptomics. (a-b) The interaction network analysis of DEGs (red rounds) and DELs (blue squares) between the QGL and MOD groups. (a) Interaction network of DEGs and DELs involved in insulin resistance. (b) Interaction network of DEGs and DELs involved in glycerolipid metabolism. The “red” edge represents positive correlation, while the “green” edge represents negative correlation. Edges width represents correlation significance, as determined by PCC. The size of rounds represents the number of DELs related to DEGs. All the LIPID-P-XXXXs in the figure are triglycerides. (c) The correlation coefficient cluster heatmap of DEGs and DELs between the QGL and MOD groups. (d) Histogram of KEGG enrichment for DEGs and DELs between the QGL and MOD groups. (e) The DEGs and DELs involved in related metabolic pathways. The yellow dashed box represents the insulin resistance process and the green dashed box represents the triglyceride biosynthesis process. The rectangle was divided into two equal parts (the left side represents DEGs or DELs in MOD vs CON; the right side represents DEGs or DELs in QGL vs MOD). PPP1R3G: protein phosphatase 1 regulatory subunit 3G; AKT: serine-threonine protein kinase; SREBP1: sterol-regulatoryelement-binding protein 1; GPAT3: glycerol-3-phosphate acyltransferase 3; FAS: fatty acid synthase; ACC: acetyl-CoA carboxylase; FA: fatty acid; G6PC: glucose-6-phosphatase; PGC-1*α*: alpha peroxisome proliferator-activated receptor gamma coactivator 1-alpha; CREB: cyclic AMP-responsive element-binding protein; CRTC2: CREB-regulated transcription coactivator 2; SIK1: salt-inducible kinase 1; and PPP1R3C: protein phosphatase 1 regulatory subunit 3C.

PCC was applied to represent the correlations between DEGs and DELs. The coexpression network analysis of DEGs and DELs (Figures 4(a) and 4(b)) and the correlation coefficient cluster heat map (Figure 4(c)) based on Pearson's correlation are shown. The DEGs and DELs between the QGL and MOD groups, such as TG, SIK1, PPP1R3C, CRTC2, PGC-1*α*, G6PC, PPP1R3G, and SREBP1 were found to be relevant to insulin resistance. While TG, SIK1, PPP1R3C, and SREBP1 were mainly enriched in triglyceride biosynthesis. DEGs involved in insulin resistance and triglyceride biosynthesis pathways are summarized in Table 3. Moreover, according to the KEGG database, DEGs and DELs are mainly enriched in insulin resistance, leishmaniasis, metabolic pathways, and systemic lupus erythematosus (Figure 4(d)). A summary of QGD regulation effects on NAFLD in the HFD rats was conducted (Figure 4(e)).

## 4. Discussion

Based on our current knowledge, the primary pathogenic driver of NAFLD is the overwhelmed ability of the liver to process excess carbohydrates and fatty acids, resulting in the accumulation of toxic lipids and hepatic steatosis. These changes then induce hepatocyte stress, injury, and death, leading to inflammation, fibrosis, and a predisposition to cirrhosis and hepatocellular carcinoma [23]. This process is closely related to abnormal lipid metabolism and insulin resistance [24]. It is reported that 69% of hyperlipidemia patients [25] and over more than three-quarters of diabetics suffer from NAFLD [26]. Huangqi-Gegen drug pair and Chenpi, the components of QGD, were found to improve insulin resistance and hyperlipidemia, respectively [19, 20]. However, the effects of QGD on NAFLD have not been reported. Herein, we conducted pharmacodynamics to evaluate the efficacy of QGD on NAFLD. Then, a multiomics analysis via integrating lipidomics and transcriptomics to elucidate the molecular mechanisms. The results showed that QGD could alleviate hyperlipidemia and hepatic lipid accumulation by improving lipid metabolism and insulin resistance in the early stage of hepatic steatosis. Thus, QGD is of positive significance in preventing the progress of NAFLD.

We found that QGD ameliorated lipid metabolism disorders at the transcriptional and metabolic levels mainly through insulin resistance and the triglyceride biosynthesis pathway (Figure 4(e)). Insulin modulates hepatic gluconeogenesis-related genes, thus controlling glucose production to ultimately maintain normoglycemia [27]. In the case of insulin resistance, insulin fails to inhibit postprandial hepatic gluconeogenesis, resulting in an increase in blood glucose. Therefore, impaired suppression of gluconeogenesis is widely considered to be a sign of compromised hepatic insulin signaling [28]. Our research indicated that QGD may improve insulin resistance via regulating the expression of CRTC2, SIK1, PPP1R3C, PGC-1*α*, G6PC, PPP1R3G, and SREBP1, which are involved in gluconeogenesis. CREB-regulated transcription coactivator 2 (CRTC2) is critical in fasting-induced hepatic gluconeogenesis. The binding of dephosphorylated CRTC2 to phosphorylated cAMP response element-binding protein (CREB) leads to an enhancement of gluconeogenic gene expression, thereby promoting gluconeogenesis [29]. Salt-induced kinase 1 (SIK1) is one of the AMP-activated kinases. PPP1R3C is one of seven genes encoding PP1 regulatory subunits. PP1's regulatory subunit and catalytic subunit (PP1C) constitute protein phosphatase 1 (PP1), which has a critical function in glucose metabolism. By now, SIK1 and PPP1R3C have emerged as regulators of glucose and lipid metabolism. Overexpression of SIK1 increases the phosphorylation level of CRTC2 at the Ser171 site [30, 31]. Silencing PPP1R3C blocked the dephosphorylation of CRTC2 and limited CRTC2 nuclear entry [32], which suppressed its binding to CREB, thereby suppressing downstream genes expression involved in gluconeogenesis, mainly including peroxisome proliferator-activated receptor *γ*coactivator-1*α* (PGC-1*α*), phosphoenolpyruvate carboxykinase (Pck1), and glucose-6-phosphatase (G6PC) [33, 34]. PGC-1*α*, which is a direct target for CREB-CRTC2 complex regulation in vivo, promotes Pck1 and G6PC induction [34]. Pck1 and G6PC are the rate-limiting enzymes of the gluconeogenesis pathway. They catalyze the transformations of oxaloacetate and glucose-6-phosphate into phosphoenolpyruvate and glucose, respectively [27]. QGD increased the expression of SIK1 while inhibiting PPP1R3C, resulting in a reduction of the CRTC2 dephosphorylated level. The transcriptions of PGC-1*α* and G6PC were subsequently downregulated. As a result, gluconeogenesis and insulin resistance were improved by QGD administration. Therefore, the PPP1R3C/SIK1/CRTC2 signal axis is supposed to be the target of QGD. Moreover, we found that PPP1R3G, which is directly phosphorylated by AKT to control glycogen synthesis and glucose homeostasis in the insulin signaling pathway, was obviously elevated in the QGD group. The upregulation of PPP1R3G may be another mechanism to improve insulin sensitivity [35].

Although insulin promotes lipogenesis by increasing sterol-regulatoryelement-binding transcription factor 1 (SREBP1) expression under physiological conditions, hepatic lipogenesis is not inhibited but increased in an insulin resistance state [36]. This may be the result of mTORC1 inducing SREBP1 expression under insulin resistance [37]. SREBP1, which promotes the expression of genes involved in the biosynthesis of fatty acids and triglycerides, particularly ACC, fatty acid synthase (FAS), glycerol-3-phosphate acyltransferase (GPAT3), and stearoyl-CoA desaturase (SCD1), is a major regulator of the lipogenic pathway [13, 38]. Overexpression of SREBP1 led to increased hepatic lipid accumulation, indicating that SREBP1 played a key role in lipogenic gene activation [39, 40]. Compared with the MOD group, QGD significantly decreased the expressions of SREBP1 and its downstream target gene GPAT3 in HFD rats. The improvement of insulin resistance could be the main reason. Another possible mechanism is the transcriptional regulation of SIK1 and PPP1R3C by QGD. In addition to modulating gluconeogenesis and insulin resistance and thus indirectly influencing SREBP1 activity, both SIK1 and PPP1R3C can directly regulate SREBP1 expression. SIK1 mediates the inhibition of SREBP1 activity. On the contrary, PPP1R3C increases SREBP1 activity [40, 41]. QGD could upregulate SIK1 while downregulating PPP1R3C. These changes inSIK1, PPP1R3C, and SREBP1 expression levels in HFD rats after QGD intervention contributed to the reduction of hepatic triglyceride biosynthesis.

Moreover, lipidomics and transcriptomics analysis showed that QGD could significantly reduce the content of carnitine and increase carnitine palmitoyltransferase 2 (CPT2) activity, compared with the MOD group. Carnitine, which is crucial for the long-chain fatty acids to transfer across the inner mitochondrial membrane, is a critical substance for fatty acid *β* oxidation. CPT2 converts acylcarnitine esters back to acyl CoA esters for subsequent *β* oxidation. However, the changes in other genes involved in this pathway were not obvious. Further study is needed to illuminate the effects of QGD on fatty acid oxidation.

## 5. Conclusion

In conclusion, the lipidomics results of this research proved QGD inhibited hepatic steatosis mainly by reducing hepatic insulin resistance and triglyceride biosynthesis. Hepatic transcriptomic analysis further revealed that QGD significantly lowered the transcription of genes involved in insulin resistance and triglyceride biosynthesis pathways. The targets of QGD were supposed to be the PPP1R3C/SIK1/CRTC2 and PPP1R3C/SIK1/SREBP1 signal axis, which still need confirmation and support from further studies. In short, our findings offer a therapeutic prospect of QGD against NAFLD with a possible mechanism to improve glycolipid metabolism.

## Figures and Tables

**Table 1:** Detailed information about the botanical compositions of Qige decoction (QGD).

Herb	Chinese name	Medicinal parts	Pharmaceutical companies	Lot number	Amount in application (g)
*Astragalus mongholicus* bunge	Huangqi	Dried root	Puning Kangmei	190201651	180
			Pharmaceutical		
			(Guangdong, China)		

*Pueraria montana* var. *Lobata* (Willd.) Maesen and S.M.Almeida ex Sanjappa and Predeep	Gegen	Dried root	Tongrentang	20190103	60
			(Beijing, China)		

*Citrus* × *aurantium L*.	Chenpi	Dried	Yangjiang Xinhui	2013001	30
		Pericarp	(Guangdong, China)		

**Table 2:** Differential lipids (DELs) in liver widely-targeted lipidomics by UPLC-MS/MS (*n* = 6, QGL vs MOD).

Index	Formula	Compounds	m/z	Ion mode	VIP	*P* value	Log2FC
LIPID-P-0016	C19H37NO5	3-Hydroxy-dodecanoyl-carnitine	360.2760	[M + H]+	1.8079	0.0759	−4.1566
LIPID-P-0027	C15H27NO4	Octenoyl-carnitine	286.2025	[M + H]+	1.2305	0.1029	−1.1566
LIPID-P-0715	C48H80NO10P	PS(22 : 2/20 : 5)	862.5598	[M + H]+	1.6372	0.0003	−1.4132
LIPID-P-0840	C51H94O6	TG(14 : 0/16 : 1/18 : 1)	820.7394	[M + NH4]+	1.0090	0.0975	−1.0180
LIPID-P-0898	C51H92O6	TG(14 : 1/16 : 1/18 : 1)	818.7237	[M + NH4]+	1.1005	0.1046	−1.3527
LIPID-P-0900	C51H92O6	TG(14 : 0/16 : 1/18 : 2)	818.7237	[M + NH4]+	1.3069	0.0637	−1.0904
LIPID-P-0974	C51H90O6	TG(14 : 1/14 : 1/20 : 2)	816.7081	[M + NH4]+	1.2559	0.0663	−1.2510
LIPID-P-0976	C51H90O6	TG(14 : 0/16 : 1/18 : 3)	816.7081	[M + NH4]+	1.3677	0.0625	−1.2017
LIPID-P-1032	C53H92O6	TG(16 : 0/16 : 1/18 : 4)	842.7237	[M + NH4]+	1.2610	0.0762	−1.2353
LIPID-P-1036	C53H92O6	TG(14 : 0/18 : 2/18 : 3)	842.7237	[M + NH4]+	1.5812	0.0190	−1.0030
LIPID-P-1081	C53H90O6	TG(14 : 0/18 : 3/18 : 3)	840.7081	[M + NH4]+	1.5392	0.0118	−1.0117
LIPID-P-1122	C55H92O6	TG(14 : 0/18 : 4/20 : 3)	866.7237	[M + NH4]+	1.6973	0.0061	−1.0558
LIPID-P-1123	C55H92O6	TG(16 : 1/16 : 1/20 : 5)	866.7237	[M + NH4]+	1.6026	0.0093	−1.2570
LIPID-P-1155	C57H94O6	TG(14 : 0/20 : 3/20 : 5)	892.7394	[M + NH4]+	1.5143	0.0247	−1.0425
LIPID-P-1176	C57H92O6	TG(18 : 1/18 : 4/18 : 4)	890.7237	[M + NH4]+	1.6034	0.0224	−1.3360
LIPID-P-1190	C57H90O6	TG(18 : 2/18 : 4/18 : 4)	888.7081	[M + NH4]+	1.5834	0.0152	−1.0594

**Table 3:** Differentially expressed genes (DEGs) involved in insulin resistance and triglycerides biosynthesis pathways.

Gene name	Description	MOD vs CON		QGL vs MOD	
		Log2FC	*P* value	Log2FC	*P* value
SIK1	Salt induced kinase 1	−2.039	1.82E-08	1.284	1.23E-07
PPP1R3C	Protein phosphatase 1 regulatory subunit 3C	0.427	0.2704	−2.135	3.51E-07
PPP1R3G	Protein phosphatase 1 regulatory subunit 3G	−2.765	0.003757	1.589	0.0107
CRTC2	CREB-regulated transcription coactivator 2	−0.086	0.627441	−1.519	9.14E-05
PGC-1*α*	Alpha peroxisome proliferator-activated receptor gamma coactivator 1-alpha	−0.283	0.528324	−1.176	0.009018125
G6PC	Glucose-6-phosphatase	0.413	0.129002	−1.771	1.54E-10
SREBP1	Sterol-regulatory element-binding protein 1	0.728	0.227177	−1.427	2.34E-05
GPAT3	Glycerol-3-phosphate acyltransferase 3	1.18	0.203196	−1.878	0.049292787

## Data Availability

The data used to support the findings of this study are included within the article.
